# Comparison of Immune Responses to Different Versions of VLP Associated Stabilized RSV Pre-Fusion F Protein

**DOI:** 10.3390/vaccines7010021

**Published:** 2019-02-15

**Authors:** Lori M. Cullen, Madelyn R. Schmidt, Gretel M. Torres, Adam A. Capoferri, Trudy G. Morrison

**Affiliations:** 1 Department of Microbiology and Physiological Systems, University of Massachusetts Medical School, Worcester, MA 01655, USA; Lori.Mcginnes@umassmed.edu (L.M.C.); Madelyn.Schmidt@umassmed.edu (M.R.S.); alongwi2@jhmi.edu (A.A.C.); 2 Program of Immunology and Microbiology, University of Massachusetts Medical School, Worcester, MA 01655, USA; Gretel.M.Torres.GR@dartmouth.edu

**Keywords:** respiratory syncytial virus, VLPs, vaccine, F protein, immune responses

## Abstract

Efforts to develop a vaccine for respiratory syncytial virus (RSV) have primarily focused on the RSV fusion protein. The pre-fusion conformation of this protein induces the most potent neutralizing antibodies and is the focus of recent efforts in vaccine development. Following the first identification of mutations in the RSV F protein (DS-Cav1 mutant protein) that stabilized the pre-fusion conformation, other mutant stabilized pre-fusion F proteins have been described. To determine if there are differences in alternate versions of stabilized pre-fusion F proteins, we explored the use, as vaccine candidates, of virus-like particles (VLPs) containing five different pre-fusion F proteins, including the DS-Cav1 protein. The expression of these five pre-F proteins, their assembly into VLPs, their pre-fusion conformation stability in VLPs, their reactivity with anti-F monoclonal antibodies, and their induction of immune responses after the immunization of mice, were characterized, comparing VLPs containing the DS-Cav1 pre-F protein with VLPs containing four alternative pre-fusion F proteins. The concentrations of anti-F IgG induced by each VLP that blocked the binding of prototype monoclonal antibodies using two different soluble pre-fusion F proteins as targets were measured. Our results indicate that both the conformation and immunogenicity of alternative VLP associated stabilized pre-fusion RSV F proteins are different from those of DS-Cav1 VLPs.

## 1. Introduction

Respiratory syncytial virus (RSV) is a significant human pathogen but, despite decades of effort, no licensed vaccines exist. RSV infections can result in severe respiratory disease in the very young, the elderly, and immunocompromised populations. This virus is a common cause of severe acute lower respiratory track disease in infants and young children worldwide [1]. Infections of this population in the US frequently result in hospitalization and in developing countries, the infections cause significant mortality [1,2]. In the elderly, the importance of this virus rivals the impact of influenza infections [3,4,5,6]. It is estimated that the virus results in 11,000 to 17,000 elderly deaths per year in the US and ten times that number of RSV associated hospitalizations [7]. The world population over the age of 60 is forecast to reach 2.1 billion, more than 20% of the population, by 2050 (World Population Aging Report 2015, United Nations). Thus, RSV infections will result in a greatly increased global public health burden in the next few decades. Mortality due to RSV infection in stem cell transplant patients is estimated to be between 6–80% [8,9]. Furthermore, RSV infections also result in significant morbidity in normal adult populations [10].

Development of an RSV vaccine has been attempted since the late 1960s, without success. One major factor contributing to the failures was the lack of appreciation for the role that the conformation of the RSV F protein plays in the stimulation of protective antibody responses. Like many viral fusion proteins, the RSV F protein is folded into a metastable, pre-fusion conformation which, upon fusion activation, refolds into a structurally very different post-fusion conformation [11,12,13,14,15]. Recently, the pre-fusion form of the F protein has been shown to be most effective at inducing optimally neutralizing antibodies (NAbs) [15,16]. However, because of the instability of the pre-fusion conformation of the F protein, most vaccine candidates, until recently, have only contained the post-fusion form of F protein [17]. Indeed, recent clinical trials using the post-fusion form of the F protein have failed [18,19].

We have developed novel virus-like particle (VLP) vaccine candidates for RSV [20,21,22,23,24]. VLPs are ideal platforms for vaccines targeting many pathogens. In contrast to soluble proteins, VLPs robustly stimulate immune responses without the addition of adjuvant [25]. Because the production of VLPs does not require viral replication, multiple antigens and different conformational forms of antigens can be assembled into VLPs, in contrast to attenuated viruses, which must remain infectious. VLPs are also safer as vaccines for many populations, such as the very young or the elderly with compromised health, compared to infectious, attenuated, or vector viruses, since they do not contain a genome and do not produce a spreading infection. 

McLellan et al. have identified mutations in the RSV F protein (DS-Cav1 mutant) that stabilize the pre-fusion conformation [16]. We have constructed VLPs containing either this stabilized pre-fusion RSV F protein or a stabilized post-fusion F protein together with the RSV G protein and have established the superiority of the pre-fusion F containing VLPs [22,23,24,26] over post-F VLPs in inducing neutralizing antibodies (NAbs) in both mice and cotton rats. However, three reports [27,28,29] indicated that soluble DS-Cav1 pre-F is still somewhat unstable and converts to the post-F form upon storage. The reported instability of DS-Cav1 F could negatively impact immune responses to the F protein, so we characterized immune responses to four alternative mutation stabilized pre-fusion F proteins. We examined their expression and assembly into VLPs, the stability of the pre-fusion conformation in VLPs, the reactivity of VLP associated pre-F proteins with anti-F monoclonal antibodies, and their induction of neutralizing antibodies following the immunization of mice. Results were compared with VLPs containing the DS-Cav1 pre-F protein. In addition, we quantified the blocking of the binding of prototype monoclonal antibodies by antibodies induced following immunization with each VLP using two different soluble pre-fusion F proteins as targets. Our results indicate that VLPs expressing different versions of stabilized pre-fusion RSV F proteins can differentially impact immunogenicity. 

## 2. Materials and Methods

### 2.1. Cells, Plasmids, and Viruses

ELL-O, Vero cells, and Hep2 cells were obtained from the American Type Culture Collection and grown in DMEM (Invitrogen. Waltham, MA, USA) supplemented with penicillin, streptomycin (Invitrogen), and 5% (Vero cells) or 10% fetal bovine serum (ELL-O, Hep2 cells) (Invitrogen). Expi293F cells, obtained from ThermoFisher/Invitrogen, were grown in Expi293 media (ThermoFisher/Gibco/Invitrogen Waltham, MA, USA). RSV, A2 strain, was obtained from Dr. Robert Finberg. Virus stocks were prepared from infected Hep2 cells as previously described [20].

**Figure 1:**
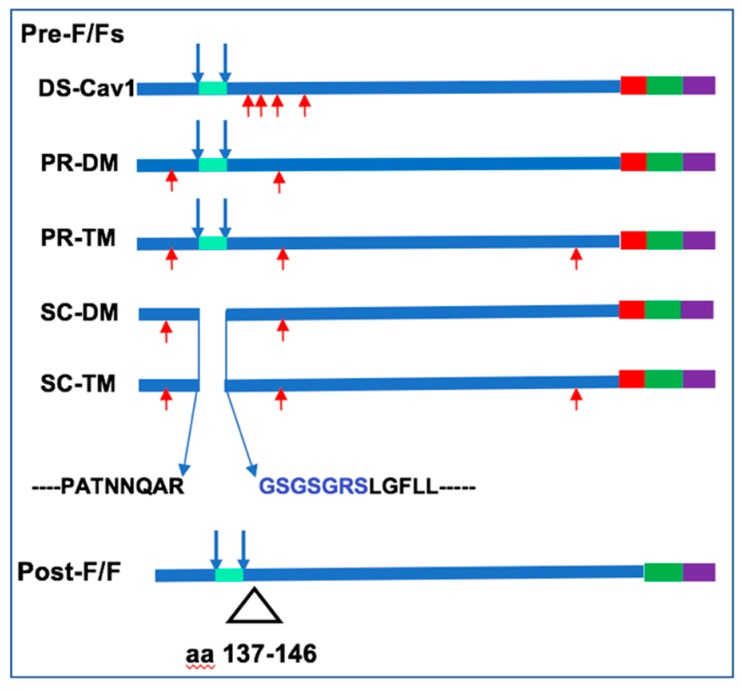
Fusion Protein Chimeras. Shown is a diagram of five different versions of mutation stabilized RSV pre-fusion F proteins used in this study. DS-Cav1 protein containing four mutations (red arrows) has been previously described [16] PR-DM and PR-TM are processed (cleaved) F proteins with two or three mutations, respectively, indicated by red arrows. SC-DM and SC-TM are uncleaved with the p27 sequence and cleavage sites deleted and a linker sequence, in blue, inserted. SC- DM and SC-TM also contained two or three mutations, respectively, indicated by red arrows. All Pre-F proteins contained the ectodomain of the RSV fusion protein fused to the foldon sequence (red), the NDV transmembrane (green), and cytoplasmic (purple) domains. Post-F/F protein contained the deletion indicated.

VLPs containing the RSV F and G protein ectodomains (from RSV stain A2) were assembled, as chimera proteins, with the Newcastle disease virus (NDV) core proteins NP and M, as previously described [20,24]. The construction, expression, and incorporation of the chimera protein NDVHN/RSVG (H/G) into VLPs have been previously described [21]. The construction, expression, and incorporation into VLPs of the stabilized pre-fusion DS-Cav1 F/F protein to generate VLP-H/G+DS-Cav1 F/F (abbreviated DS-Cav1 VLPs), and the stabilized post-fusion F protein to create VLP-H/G+post-F/F (abbreviated post-F VLPs), have been previously described [24]. Chimera proteins containing alternative versions of the pre-fusion F protein were constructed by introducing mutations into the wild-type F/F chimera. PR-DM F/F and PR-TM F/F contained mutations N67I, S215P, or N67I, S215P, and D486N, respectively. SC-DM F/F and SC-TM F/F both had deletions of the p27 sequence, including the two cleavage sites combined with the insertion of a linker sequence GSGSGRS, as diagramed in Figure 1. In addition, SC-DM F/F and SC-TM F/F had two (N67I, S215P) or three (N67I, S215P, D486N) amino acid substitutions, respectively.

The constructions of genes encoding the soluble pre-F protein and the soluble post-F protein have been previously described [24,26].

### 2.2. Antibodies

RSV F monoclonal antibody clone 131-2A (Millipore MAB8599) was used in RSV plaque assays. Murine monoclonal antibodies mAb1112 and mAb1243 (generous gifts of Dr. J. Beeler), and human mAb D25, mAb AM14, and mAb motavizumab (generous gifts of Dr. J. McLellan), were used to verify F protein conformation in ELISA analysis of VLPs and soluble F proteins, and for antibody blocking experiments. Palivizumab used for antibody blocking experiments was the generous gift of Dr. Jorge Blanco. The anti-RSV F protein HR2 antibody and anti-NDV F-tail antibody used for Western Blots are polyclonal antibodies specific to the HR2 domain of the RSV F protein or the cytoplasmic tail of NDV F protein [20]. The anti-RSV G protein antibody is a polyclonal antibody raised against a peptide containing G protein amino acids 180–198 (ThermoFisher Waltham, MA, USA). Secondary antibodies against goat, mouse, and rabbit IgG were purchased from Sigma. A secondary antibody against human IgG was purchased from Southern Biotech. 

### 2.3. VLP Preparation, Purification, and Characterization 

The conformation of the F protein in the VLP preparations was verified by reactivity to mAbs. The characterization of purified preparations of Pre-F/F VLPs and Post-F/F VLPs has been previously published [24,26]. For preparations of VLPs to be used as immunogens (abbreviated as DS-Cav1 VLPs, post-F VLPs, PR-DM VLPs, PR-TM VLPs, SC-DM VLPs, SC-TM VLPs), ELL-0 cells growing in T-150 flasks were transfected with cDNAs encoding the NDV M protein, the NDV NP, the chimera protein H/G, and one of the five Pre-F/F proteins or the Post-F/F protein, as previously described [20]. At 24 h post-transfection, heparin (Sigma) was added to the cells at a final concentration of 10 /mL to inhibit the rebinding of released VLPs to cells. At 72, 96, and 120 h post-transfection, cell supernatants were collected and VLPs purified by sequential pelleting and sucrose gradient fractionation, as previously described [30]. Briefly, cell debris from the supernatant was removed by centrifugation at 5000 rpm (Sorvall GSA SLA-1500 rotor), and VLPs in the supernatant were pelleted by centrifugation in a Type 19 Rotor (Beckman) at 18,000 rpm for 12 h. The resulting pellet was resuspended in TNE buffer (25 mM Tris-HCl, pH 7.4, 150 mM NaCl, and 5 mM EDTA), dounce homogenized, and layered on top of a discontinuous sucrose gradient (2 mL 65% sucrose and 4 mL 20% sucrose). The gradients were centrifuged in an SW 28 rotor (Beckman Brea, CA, USA) at 24,000 rpm for 6 h and the fluffy layer at the 20–65% sucrose interface, containing the VLPs, was collected, mixed with two volumes of 80% sucrose, placed on top of a 1 mL layer of 80% sucrose in a SW41 Beckman centrifuge tube, and then over layered with 3.5 mL of 50% sucrose and 2 mL of 10% sucrose. The gradients were centrifuged to equilibrium for 18 h at 38,000 rpm. The VLPs, all of which floated up into the sucrose to the same density, were collected and concentrated by centrifugation in an SW50.1 rotor for 16 h at 38,000 rpm. All sucrose solutions were w/v and dissolved in TNE buffer and all centrifugations were conducted at 4 °C.

The characterization of purified preparations of all VLPs was completed as previously described for Pre-F VLPs and Post-F VLPs [24,26]. The conformation of the F protein in the VLP preparations was verified by reactivity to mAbs (as in Figure 3 and previously described [24,26]). Protein concentrations of VLP associated F proteins were calculated from a standard curve generated with a parallel western blot of a purified soluble F protein of a known concentration.

### 2.4. Preparation of Soluble F Proteins

Expi293F cells were transfected with cDNAs encoding the soluble DS-Cav1 pre-F protein, the soluble SC-TM pre-F protein, and the soluble post-F protein. At six days post transfection, total cell supernatants were collected and cell debris removed by centrifugation. Soluble polypeptides were then purified on columns using the His tag and then the strep tag, as previously described [24,26]. Our soluble DS-Cav1 pre-F protein and soluble SC-TM pre-F protein efficiently bind to pre-fusion specific mAbs AM14 and D25. The soluble post-F does not bind AM14 or D25, but does bind motavizumab, a site II antibody. Validation of these soluble proteins is described in Blanco, et al. [26].

### 2.5. Detection of Cell Surface Protein by Surface Biotinylation 

ELL-0 monolayers were grown in 35 mm plates and transfected with cDNAs encoding the F/F proteins or F/F and H/G proteins. After 48 h, the monolayers were washed three times with PBS-CM (PBS with 0.1 mM CaCl_2_ and 1 mM MgCl_2_). PBS-CM containing 0.5 mg/mL sulfo-NHS-SS-biotin (Pierce Biotechnology, Waltham, MA, USA) was added and cells were incubated for 40 minutes at 4 °C. Unbound biotin was absorbed with 2 mL DMEM containing fetal calf serum (10%) and cells were washed three times with PBS and lysed with RSB lysis buffer (0.01 M Tris-HCl [pH 7.4], 0.01 M NaCl, 1.5 mM MgCl_2_) containing 1% Triton X-100, 0.5% sodium deoxycholate, 2.5 mg of N-ethyl maleimide per mL, and 0.2 mg of DNase per mL. Lysates were incubated for 1 h at room temperature or overnight at 4 °C with neutravidin-agarose (Pierce), containing 0.3% SDS, that had been washed with PBS containing 0.5% tween-20 and 5 mg/mL BSA and then with PBS containing 0.5% Tween-20 and 1 mg/mL BSA. Precipitates containing biotinylated proteins were recovered by centrifugation, washed three times with PBS containing 0.5% Tween-20 and 0.4% SDS, resuspended in gel sample buffer (125 mM Tris-HCl, pH 6.8, 2% SDS and 10% glycerol) with 0.7M β-mercaptoethanol, and resolved by polyacrylamide gel electrophoresis. F proteins in the precipitate were detected by Western analysis using an anti-NDV F tail antibody. 

### 2.6. Measures of Relative Binding of Mab to Purified VLPs

VLPs containing equivalent amounts of F protein (determined by Western blots) were added to microtiter wells and incubated for 2–4 h at room temperature. Different dilutions of different mAb were added to the wells, incubated for 2 h, and removed, and the wells were washed with PBS. The mAbs were then removed, and the plate was washed in PBS and incubated with goat anti-human IgG coupled to HRP for 2 h at room temperature. Bound HRP was detected using TMB (3,3’5,5’-tetramethylbenzidin, ThermoFisher34028, Waltham, MA, USA) and the reaction was stopped with 2N sulfuric acid. Color was read in the SpectraMax Plus Plate Reader (Molecular Devices) using SoftMax Pro software. Results are expressed as optical density (OD).

### 2.7. Determination of Stability of Pre-Fusion F Conformation

For determination of the stability of pre-fusion F conformation in VLPs, VLPs with equivalent amounts of F protein were incubated at different temperatures, different pHs, or different salt concentrations for one hour. The VLPs were then bound to microtiter wells overnight at 4 °C. The wells were incubated with PBS-2% BSA and then incubated with mAb D25 for one hour, and the binding of mAb was detected using anti-human IgG coupled to HRP. Bound HRP was detected as described above. 

### 2.8. Determination of Total Anti-F Protein IgG in Sera

For the determination of anti-pre-F protein or post-F protein IgG antibody levels, wells of microtiter plates (ThermoFisher/Costar, Waltham, MA, USA) were coated with either purified soluble DS-Cav1 F protein or soluble post-fusion F protein (30 ng/well) and incubated overnight at 4 °C, before being blocked with 2% BSA for 16 h. Different dilutions of sera, in PBS-2% BSA and 0.05% Tween, were added to each well and incubated for 2 h at room temperature. Wells were then washed with PBS, incubated with sheep anti-mouse antibody coupled to HRP (Sigma A5906, St. Louis, MO, USA), and incubated for 1.5 h at room temperature. Bound HRP was detected using TMB (3,3’5,5’-tetramethylbenzidin, ThermoFisher34028) and the reaction was stopped with 2N sulfuric acid. Color was read in the SpectraMax Plus Plate Reader (Molecular Devices, San Jose, CA, USA) using SoftMax Pro software. Amounts of anti-pre-F or anti-post-F IgG (ng/mL) in each dilution were calculated using a standard curve generated in parallel using defined amounts of purified murine IgG. 

### 2.9. RSV Plaque Assays, Antibody Neutralization, and Antibody Blocking

RSV was grown in Hep2 cells, and RSV plaque assays were accomplished on Vero cells as previously described [24,26]. Antibody neutralization assays in a plaque reduction assay have been previously described [24]. Neutralization titer was defined as the reciprocal of the dilution of serum that reduced the virus titer by 50%.

To measure the ability of polyclonal sera to block the binding of mAbs, different dilutions of sera were diluted in PBS-1% BSA, and then incubated for one hour at room temperature in wells of Ni-coated microtiter plates (Pierce/ThermoFisher) containing pre-bound 50 ng soluble DS-Cav1 pre-F protein or soluble SC-TM pre-F protein. Ni-coated plates were used in order to bind the soluble pre-F proteins via the histidine tag at the carboxyl terminus of the protein and thus orient the protein in the well with the apex of the molecule projecting upwards, as in virus particles. After removal of the serum, the wells were incubated with 200 ng/mL of purified mAb diluted in PBS-1% BSA for 10 min at room temperature. The mAb was then removed, and the plate washed in PBS and incubated with goat anti-human IgG coupled to HRP. After incubation for one hour at room temperature, the bound HRP was detected as in ELISA assays. The total anti-pre-F IgG in the different serum dilutions used for mAb blocking was determined using a standard curve of purified murine IgG (Southern Biotech. Pittsburg, PA, USA) in order to measure the ng of the serum anti-pre-F antibody that blocked binding of the mAb.

### 2.10. Animals, Animal Immunization, and RSV Challenge 

Mice, four-week-old BALB/c, from Taconic laboratories, were housed (groups of five) under pathogen-free conditions in micro isolator cages at the University of Massachusetts Medical Center animal quarters. Protocols requiring open cages were accomplished in biosafety cabinets. BALB/c mice, in groups of five animals, were immunized by intramuscular (IM) inoculation of VLPs containing 7 μg F protein in 0.05 mL of TNE (50 mM Tris-HCl, pH 7.4, 150 mM NaCl, 5 mM EDTA) containing 10% sucrose. Boosts contained 3 µg of VLP F protein. For infections with RSV, the animals were lightly anesthetized with isoflurane and then infected by intranasal (IN) inoculation of 50 μL of virus (1 × 10^6^ pfu). All animal procedures and infections were performed in accordance with the University of Massachusetts Medical School IACUC approved protocols; IACUC docket # A1982-17, approved 9-13-2017 to 9-12-2020.

### 2.11. Statistical Analysis

Statistical analyses (student *t* test and one-way ANOVA) of data were accomplished using Graph Pad Prism 7 software.

## 3. Results

### 3.1. Incorporation of Pre-fusion F Proteins Into VLPs

VLPs containing the RSV proteins are based on the Newcastle disease virus core proteins NP and M protein and contain the RSV F and G proteins [20]. The RSV F and G proteins are assembled into these VLPs as chimera proteins containing sequences of the ectodomains of the RSV F protein or G protein fused to the transmembrane (TM) and cytoplasmic (CT) domains of the NDV F or HN protein, respectively. To prepare VLPs containing alternative pre-fusion F proteins, we constructed four different versions of mutation stabilized pre-fusion F proteins, described by Krarup, et al and based on their analysis of the structure of their pre-fusion F proteins [27]. Wild-type F protein is cleaved during intracellular transport at two sites releasing a p27 peptide sequence. Two of these mutants contained the wild-type cleavage sites and either two (N67I, S215 P) or three point (N67I, S215P, D486N) mutations to generate cleaved F protein PR-DM (processed, double mutant) and PR-TM (processed, triple mutant), respectively. The other two mutants had the cleavage site sequences and the intervening p27 sequence replaced with a seven-amino acid GS rich linker sequence (Figure 1). In addition, two (N67I, S215P) or three (N67I, S215P, D486N) amino acid changes were introduced into the ectodomain sequences to generate SC-DM (uncleaved, double mutant) and SC-TM (uncleaved, triple mutant) F proteins, respectively (Figure 1). For assembly into VLPs, the sequences encoding the ectodomains of these F proteins were fused to the sequences encoding the foldon sequence [31], as well as the transmembrane (TM) and cytoplasmic (CT) domains of the NDV F proteins to generate RSVF/NDVF chimera proteins, PR-DM F/F, PR-TM F/F, SC-DM F/F, and SC-TM F/F (Figure 1). Chimera protein DS-Cav1 pre-fusion F/F, which is cleaved and contains mutations different from the PR and SC mutant proteins (Figure 1), has been previously described as has post-fusion F/F chimera protein [23,24]. 

**Figure 2:**
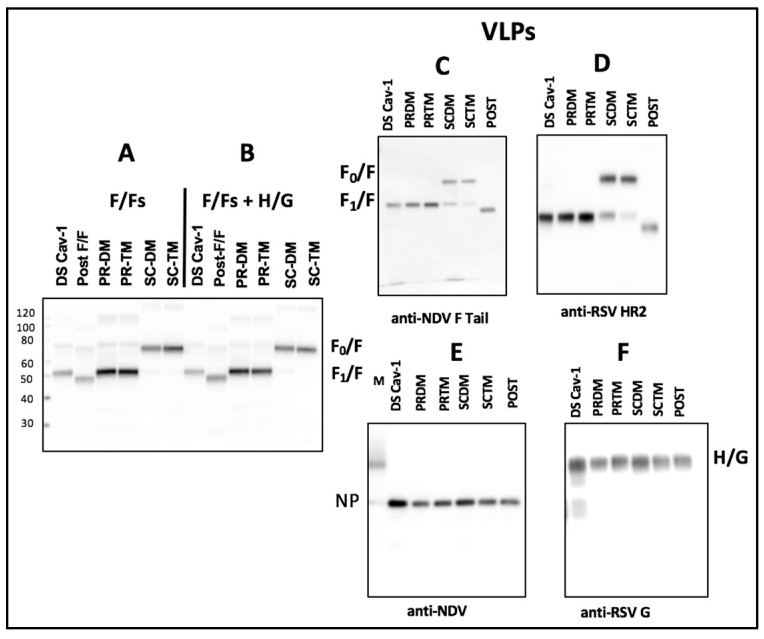
Expression of chimera proteins and VLP content: Panel **A**: Shown is a western blot of cell surface biotinylated RSV F proteins detected on the surfaces of ELL-0 cells (1 × 10^5^ cells) transfected with each of the cDNAs encoding the chimera proteins described in Figure 1. F proteins were detected using the anti-RSV HR2 antibody. Panel **B**: Shown is a western blot of biotinylated RSV F proteins detected on surfaces of cells transfected as in panel A with the addition of a cDNA encoding the H/G chimera. Panel **C**–**F**: Western blots of proteins in purified VLPs adjusted for similar F protein content based on results shown in Figure S1, Supplementary Materials. Panel **C**, F/F proteins detected with anti-NDV F tail antibody; panel D, F/F proteins detected with anti-RSV HR2 antibody; panel **E**, NDV NP protein content; panel **F**, H/G protein content. Anti-RSV does not detect F protein. F0, uncleaved F/F chimera; F1, cleaved F/F chimera; H/G, NDV HN/RSVG protein chimera; NP, NDV NP protein; M, marker proteins.

The total cell surface expression in avian cells of the four chimera proteins was compared with that of DS-Cav1 F/F and post-F/F in the absence or presence of the expression of the H/G (NDV HN/RSV G protein) chimera protein (Figure 2, panels A, B). The four PR and SC mutant proteins were more robustly expressed than the DS-Cav1 F/F or post-F/F proteins (panel A), as previously reported [27]. Co-expression of H/G did not alter the expression levels of any of the F chimeras (Figure 2, panel B).

Purified VLPs containing the F proteins were prepared and purified as previously described [30]. All VLPs contained the same H/G chimera and one of the F/F chimera proteins. VLPs containing PR-DM F/F, PR-TM F/F, SC-DM F/F, or SC-TM F/F will be referred to as PR-DM, PR-TM, SC-DM, or SC-TM VLPs, respectively. The protein content of purified large-scale stocks of VLPs was initially assessed by Western blots shown in Figure S1, Supplementary Materials and stocks adjusted for equivalent F protein content. Figure 2, panels C, D, E, and F, show the protein content of these adjusted VLP stocks. Quantitation of the content of the F/Fs, H/G, and NP relative to that in DS-Cav1 VLPs is shown in Table S1 Supplementary Materials. The DS-Cav1 VLPs contained higher amounts of NP and H/G relative to the other VLPs, suggesting some differences in the efficiency of incorporation of the DS-Cav1 F into VLPs compared to the other VLPs. This difference may relate to the lower efficiency of expression of the DS-Cav1 F protein shown in panel A and B. The F protein in Post-F/F VLPs is not detected as well with the anti-RSV HR2 antibody compared to the anti-NDV F tail antibody. 

### 3.2. Relative Binding of Monoclonal Antibodies to Different VLPs

**Figure 3:**
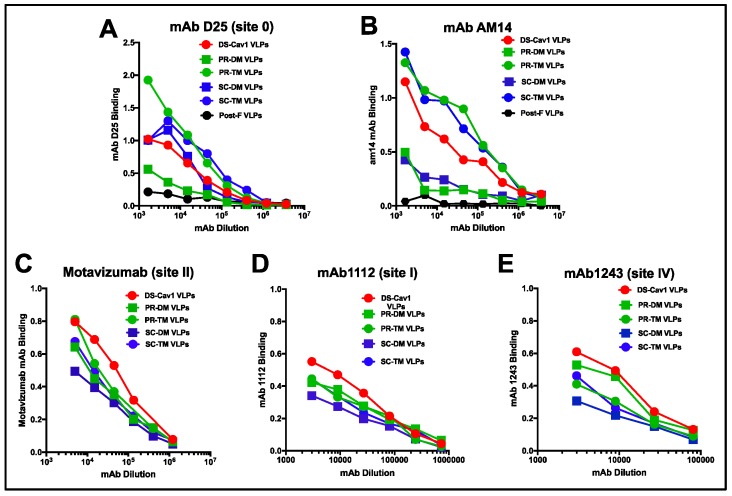
Relative binding of monoclonal antibodies to VLPs: Binding, measured as optical density, of decreasing amounts of pre-fusion F specific monoclonal antibodies to VLPs is shown in panels **A** (mAb D25, site 0) and **B** (mAb AM14, a trimer specific antibody). Binding to VLPs of mAb specific to sites common to both pre- and post-fusion F proteins are shown in panels **C**–**E**. Panel C: motavizumab (site II); Panel D: mAb 1112 (site I); Panel E: mAb 1243 (site IV). Equivalent amounts of F protein in VLPs were bound to microtiter wells. Increasing dilutions of the antibodies were added to the wells and binding of the mAb was detected using anti-human (panels **A**, **B**, and **C**) or anti-mouse (panels **D** and **E**) IgG coupled to HRP. Results are representative of three separate experiments. A separate experiment is shown in Figure S2, Supplementary Materials, along with the binding of mAb to Post-F VLPs.

To assess the retention of the pre-fusion conformation of the F proteins in VLPs, the binding to the VLPs of two pre-fusion F specific monoclonal antibodies (mAbs), D25 (site 0, at the apex of the pre-fusion F protein [15,16]) and AM14, a trimer specific, pre-fusion specific antibody [32], was measured (Figure 3). For this experiment, equivalent micrograms of VLPs associated F proteins were bound to microtiter plates and then incubated with increasing dilutions of each mAb. All pre-F containing VLPs bound both AM14 and D25, indicating that the F proteins in these VLPs retained at least some F protein in the pre-fusion conformation. However, D25 antibody binding showed unexpected differences. While DS-Cav1 VLPs and SC-DM VLPs bound mAb D25 similarly (panel A), PR-TM and SC-TM VLPs bound this antibody at higher levels and PR-DM VLPs bound this antibody at much lower levels than DS-Cav1 VLPs. 

The binding of AM14 also showed surprising differences (Figure 3, panel B). The PR-TM and SC-TM VLPs bound this antibody at much higher levels than the DS-Cav1 VLPs, while the PR-DM and the SC-DM VLPs bound this antibody poorly. That SC-DM very poorly bound mAb AM14 but bound D25 at levels similar to DS-Cav1 VLPs suggests that this epitope in this F protein may be different from DS-Cav1. The poor binding of both mAbs D25 or AM14 to PR-DM VLPs, which was only slightly increased from binding to the negative control, the post-F VLPs, suggests that the majority of this F protein may not be in a pre-fusion conformation or, alternatively, it may be in a conformation intermediate between pre-F and post-F. Thus, the differences in mAb binding to the different VLPs suggest that the VLP associated SC and PR pre-fusion F proteins have conformational differences from DS-Cav1.

Panels C, D, and E show the relative binding of monoclonal antibodies specific to sites common to both pre-fusion and post-fusion forms of the F protein, motavizumab (site II), mAb 1112 (site I), and mAb 1243 (site IV). The site II and I mAb binding to all VLPs was similar, although binding to DS-Cav1 VLPs was reproducibly slightly increased over the other VLPs. Interestingly, the antibody to site IV showed more variation in binding, suggesting some differences in the conformation of this mAb epitope in the different pre-fusion F proteins. 

### 3.3. Stability of the Pre-Fusion F Proteins in the VLPs

**Figure 4:**
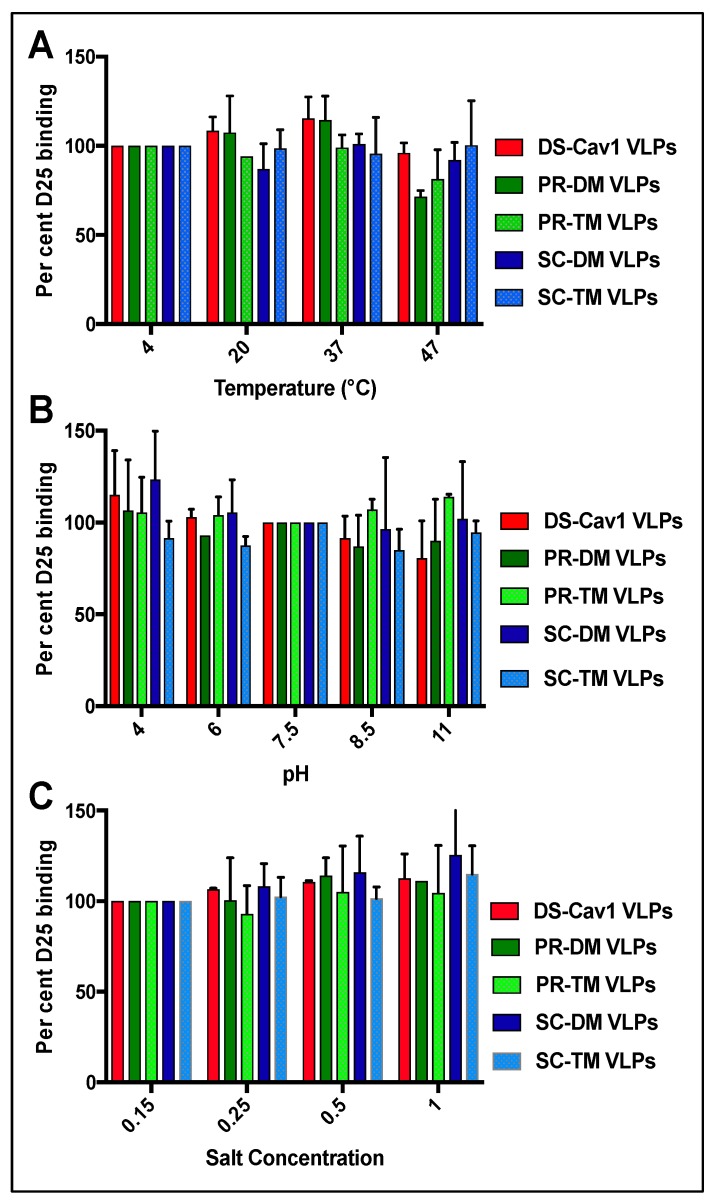
Stability of pre-fusion F conformation in VLPs. Equivalent amounts of F protein in VLPs were incubated for one hour at the temperatures, pH, or salt concentrations indicated. The binding of mAb D25 to the VLPs was quantified using anti-human IgG coupled to HRP. Results are presented as mAb 25 bound at each condition as a percent of the binding at 4 °C (panel **A**), or pH 7.5 (panel **B**), or 0.15 M salt (Panel **C**). Results are the mean with standard deviations of three separate determinations. Differences were not statistically significant (student *t* test).

Because of reports of the instability of the soluble DS-Cav1 pre-fusion F protein [27,28,29], we characterized the stability of the pre-fusion conformation of all these chimera proteins in VLPs (Figure 4) at different temperatures (Panel A), pH conditions (Panel B), and salt concentrations (Panel C). The degree of retention of the pre-fusion conformation was determined as the percent of mAb D25 binding relative to binding to untreated controls. Figure 4 shows that none of the VLPs lost statistically significant reactivity to mAb D25 after incubation in different conditions. Indeed, some treatments somewhat increased the mAb D25 binding. Thus, in contrast to the reported instability of the soluble form of the DS-Cav1 pre-fusion F protein, insertion of this chimera F protein into VLP membranes may stabilize the pre-fusion conformation of this protein. In addition, the pre-fusion conformation of the other four pre-fusion F proteins was also stable and may be stabilized by their insertion into the VLP membrane. 

### 3.4. Induction of Neutralizing Antibodies in Mice

**Figure 5:**
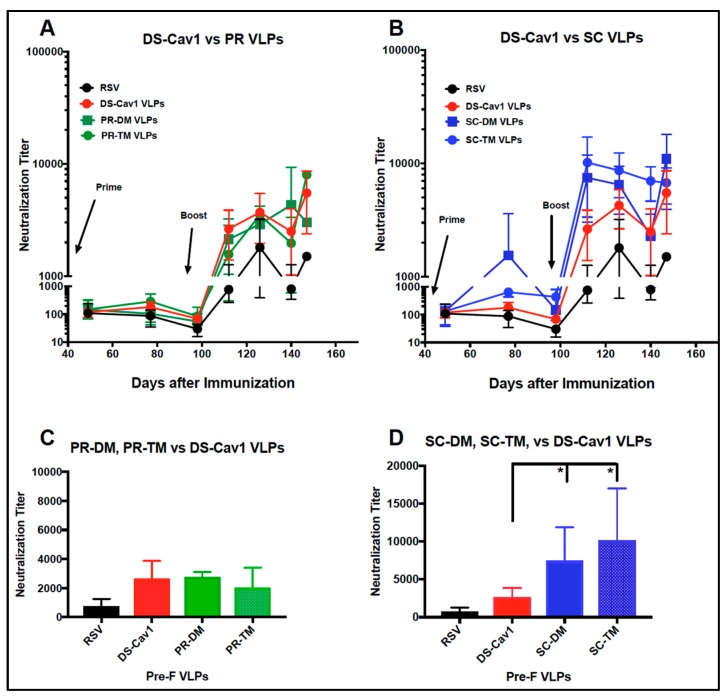
Neutralization titers in sera after VLP immunization. Sera obtained from each group of mice at each time point after the VLP prime immunization were pooled and NAbs titers determined in a classical plaque reduction assay. Panel A compares titers obtained with DS-Cav1 VLP sera with titers obtained with PR-DM VLP and PR-TM VLP sera. Panel B compares titers using sera from SC-DM and SC-TM VLPs with DS-Cav1 VLP sera. Panel C compares titers obtained at two weeks post-boost with the PR VLP sera, DS-Cav1 sera, and RSV infection with statistical analysis between groups. Panel D compares titers at two weeks post-boost with DS-Cav1 VLPs and SC VLPs and RSV infection with statistical analysis between groups. NAbs titers increase at day 150 due to boost effects of the RSV challenges of mice at day 147, four days before sacrifice. Results are the mean with standard deviation of three to six separate determinations. Comparison of titers obtained with DS-Cav1 and SC-VLP sera at two weeks post-boost was assessed by one-way ANOVA. There were no differences between DS-Cav1 and PR-VLP sera. * *p* < 0.05.

We next asked if the VLPs containing different versions of stabilized pre-fusion F protein induced similar levels of neutralizing antibodies in mice. Groups of mice were primed with intramuscular injection (IM) of 7 µg VLP associated F protein and then boosted at day 100 with 3 µg of the same VLP. Figure 5 shows the neutralizing antibody (NAbs) titers of pooled sera from each group at the indicated time after the prime immunization. Panel A compares titers obtained after immunization with PR VLPs or DS Cav-1 VLPs; panel B compares them with SC VLPs and DS Cav-1 VLPs. There were no statistically significant differences between titers obtained by immunization with the PR VLPs and DS Cav-1 VLPs (Figure 5, panel C). In contrast, both the SC VLPs induced two- to three-fold higher NAbs titers than the DS-Cav1 VLPs both after the prime and after the boost (panel B). The titers two weeks after the boost are shown in panel D. The titers obtained with SC VLP immunizations were statistically significantly higher than titers obtained with the DS- Cav1 VLPs. 

### 3.5. Total Anti-F Antibody Titers

**Figure 6:**
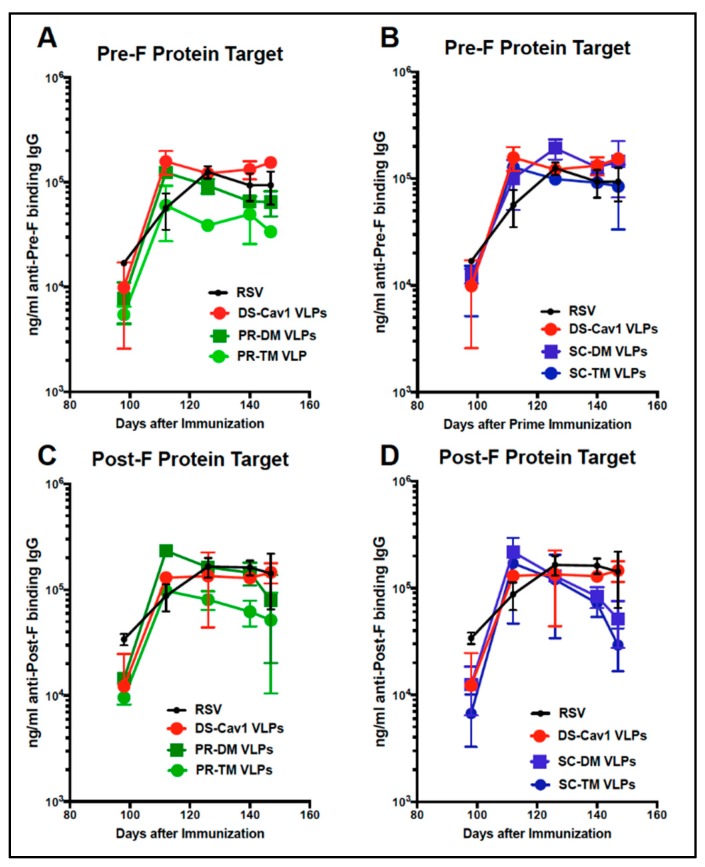
Total anti-pre-F and post-F binding IgG in sera after immunization with different VLPs. Total anti-pre-F binding (Panels **A**, **B**) and post-F binding (Panels **C**, **D**) IgG in pooled sera at each time point after the boost immunization was determined by ELISA using soluble DS-Cav1 protein (panels **A** and **B**) or soluble post-F protein (panels **C** and **D**) as the target antigen. Panels A and C compare titers in DS-Cav1 VLP sera with those in sera from PR-DM or PR-TM VLP sera. Panels B and D compare titers in DS-Cav1 VLP sera with those in sera from SC-DM or SC-TM VLP sera. Shown are the mean and standard deviations of three separate determinations. There were no statistically significant differences between titers at each time point (student *t* test).

To determine if the differences in NAbs titers could be due to differences in total anti-F antibodies induced by the different VLPs, the total anti-pre-F or anti-post-F IgG induced was measured by ELISA. Titers of antibodies (ng/mL) that bound soluble pre-fusion F protein (Figure 6, panels A, B) or soluble post-fusion F protein (Panels C, D) were measured. Figure 6, panels A and C, shows anti-F IgG titers with times after the prime and boost comparing sera from PR VLPs with DS-Cav1 VLP immunizations. Panel B and D shows titers in sera from both SC VLPs compared to DS-Cav1 VLPs. There were no statistically significant differences in anti-F protein antibody titers that bound either the soluble pre-F or post-F targets in sera induced by any of the pre-fusion F/F VLPs. 

### 3.6. Relative Concentration of D25 and AM14 Blocking Antibodies in Sera

**Figure 7:**
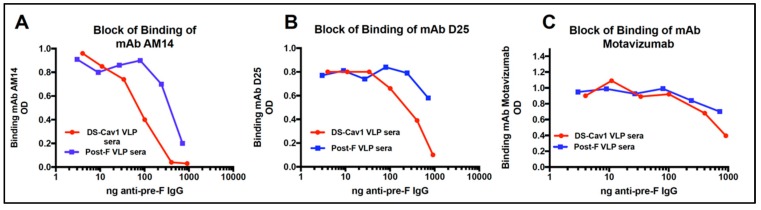
Amounts of polyclonal antibody that blocks binding of representative mAb. Shown is the ability of anti-pre-F IgG in DS-Cav1 VLP sera and in Post-F VLP sera to block binding of mAb to soluble DS-Cav1 targets. Results are plotted as binding of the 200 ng of mAb in the presence of increasing amounts (ng/mL) of anti-pre-F IgG in each pooled serum. Binding of mAb is detected using anti-human IgG coupled to HRP, as described in Materials and Methods. Panel A shows the inhibition of binding of mAb AM14 with different amounts of anti-pre-F IgG. Panel B shows inhibition of binding of mD25. Panel C shows inhibition of binding of motavizumab. Red, DS-Cav1 VLP sera; Blue, Post-F VLP sera.

That both the SC VLPs induce higher neutralization antibody titers than DS-Cav1 VLPs, but total anti-F IgG antibodies are similar, suggests that antibodies induced by the SC VLPs differ in epitope specificities from those induced by DS-Cav1 VLPs. To test this hypothesis, the concentration of anti-pre-F binding IgG antibodies in the different sera that block binding of representative pre-fusion specific mAb to soluble pre-fusion F protein was measured. For these experiments, the approach was first validated using sera from Post-F and DS-Cav1 VLP immunized mice, as shown in Figure 7, panels A-C. A soluble pre-F protein target (soluble DS-Cav1) was bound to Ni-coated microtiter plates to ensure that the target F protein bound preferentially through the polyhistidine tag at its carboxyl terminus in order to mimic presentation of the protein as in a virus or VLPs. The binding of pre-fusion, trimer specific mAb AM14 (panel A); pre-fusion specific site 0 mAb D25 (panel B); and site II specific mAb motavizumab (panel C) to a soluble DS-Cav1 F protein target in the presence of increasing concentrations of DS-Cav1 VLP induced anti-pre-F IgG or Post-F VLP induced anti-pre-F IgG in sera was measured. The figure shows that approximately 95 ng/mL of total anti-pre-F protein IgG in the DS-Cav1 VLP sera blocked 50% of AM14 binding, while 675 ng/mL of total anti-pre-F IgG in the post-F VLP sera was required to block 50% of the AM14 binding. Thus, Post-F VLP sera contained a much lower concentration of antibodies that will block mAb AM14 binding than sera induced by DS-Cav1 VLPs. Panel B shows that approximately 425 ng/mL of anti-pre-F IgG in DS-Cav1 VLP sera was required to block 50% of binding of mAb D25, while an estimated 2333 ng/mL of anti-F IgG in Post-F VLP sera was required to block 50% of mAb D25 binding. Again, DS-Cav1 VLP sera had much higher concentrations of anti-pre-F antibodies that blocked D25 binding than sera from Post-F VLP immunization. Panel C shows that similar amounts of anti-pre-F binding IgG in DS-Cav1 VLP sera and in Post-F VLP sera were required to block 50% of motavizumab binding.

This experiment has been repeated in two other completely different experiments with separate groups of five mice immunized with different preparations of the DS-Cav1 and Post-F VLPs and the results from all three experiments are shown in Table 1. 

In these experiments, using sera from four weeks post-boost, 65–138 ng/ mL of anti-pre-F IgG in the DS-Cav1 VLP sera was required to block 50% binding of mAb AM14, while 675–1500 ng/mL of anti-pre-F IgG in post-F VLP sera was required. Using mAb D25, 425–550 ng/mL of anti-pre-F IgG in DS-Cav1 VLP sera was required to block 50% of the binding of D25, while post-F VLP sera required 2333–8333 ng/mL to block 50% of the binding of mAb D25. Again, the concentration of antibodies in DS-Cav1 VLP sera that blocked the binding of mAb D25 was much higher than in post-F VLP sera. 

**Figure 8:**
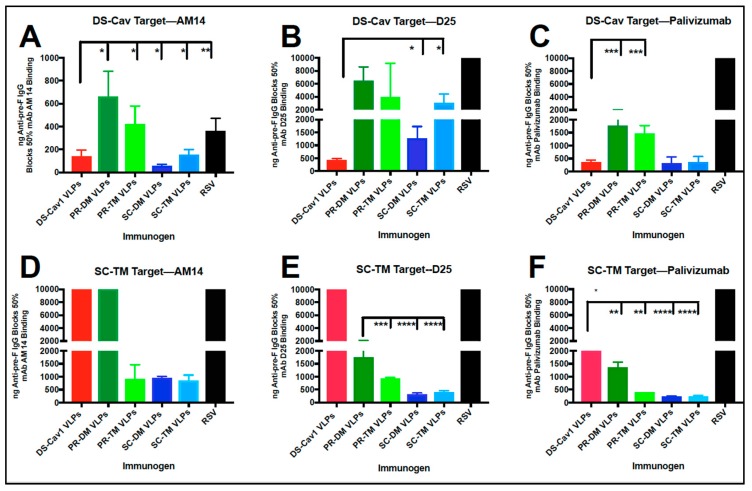
Blocking of the binding of representative mAb by sera from PR, SC, and DS-Cav1 VLP immunizations. Shown are concentrations (ng/mL) of anti-pre-F IgG in pooled sera obtained at four weeks after boost immunizations with all pre-fusion F VLPs and with RSV infection that blocked 50% binding of mAb AM14 (panel **A**), D25 (panel **B**), and palivizumab (panel **C**) to the soluble DS-Cav1 target protein. Panels **D**, **E**, and **F** show concentrations (ng/mL) of anti-pre-F IgG in pooled sera that blocked binding of mAbs AM14, D25, and palivizumab, respectively, to the soluble SC-TM target protein. The results are the mean of at least three separate determinations, with standard deviations indicated. Comparison of values with those of DS-Cav1 VLP sera was assessed by one-way ANOVA. Panel E DS-Cav1 VLP sera did not block binding of D25, thus values were compared to PR-DM VLP sera; **** *p* < 0.00005 *** *p* < 0.0005, ** *p* < 0.005, * *p* <0.05. Values at or near 10,000 ng/mL indicate failure of the sera to block binding of the tested mAb. Values at or above 2000 ng/mL indicate sera that only very weakly blocked binding. These values were quite variable from experiment to experiment, as indicated by the large standard deviation, and thus were not included in ANOVA analysis.

In parallel with experiment three shown in Table 1, the concentrations of anti-pre-F IgG in sera from the PR and SC VLP immunizations required to block 50% of the binding of AM14 and D25 were determined. Values obtained with pooled sera obtained at four weeks post-boost are shown in Figure 8, panels A-C, while the values obtained from sera obtained at two, four, and seven weeks post-boost are shown in Figure S3, Supplementary Materials. 

The sera that blocked mAb AM 14 binding at concentrations comparable to DS-Cav1 VLP sera were those from SC-DM VLP and SC-TM VLP immunizations (73 +/− 12 ng/mL and 150 +/− 40 ng/mL, respectively) (Figure 8A). Sera from the PR VLP immunizations required 650 +/− 100 ng/mL (PR-DM) and 420 +/− 100 ng/mL (PR-TM) anti-pre-F IgG to block 50% of the binding of mAb AM14. None of the VLPs induced sera that blocked D25 binding at levels comparable to DS-Cav1 VLP sera. Again, SC-DM sera contained the highest concentration of mAb D25 blocking antibodies (Figure 8B). 

The sera from Post-F VLP immunizations (Figure S3A,B, Supplementary Materials) or from RSV immunized animals (Figure 8A,B) were either not able to block or very weakly blocked the binding of mAb D25 and AM14.

Competition of polyclonal sera with binding of palivizumab has been commonly used to assess the effectiveness of responses to RSV vaccine candidates [33]. It has, however, become clear that the results are not necessarily predictive of the success of the vaccine candidate in animals or humans [18,19]. However, because of its common use in vaccine candidate assessment, we included quantification of the concentration of anti-pre-F antibodies in all sera that blocked palivizumab binding. The results are shown in Figure 8, panel C. The sera from animals immunized with all five pre-fusion F containing VLPs blocked palivizumab binding to varying degrees, with DS-Cav1 and the SC VLPs inducing the highest concentration of blocking antibodies. Thus, the relative concentrations of antibodies induced by the five-different pre-fusion F containing VLPs that will block the binding of three different monoclonal antibodies varies significantly, indicating that the pools of antibodies induced by the different VLPs are not the same. 

The target for the serum antibody blocking of mAb binding described above was soluble DS-Cav1 F protein. The mutations in the PR and SC mutant F proteins contained single amino acid changes in regions of the F protein previously identified as forming or near the site 0 epitope (amino acids 61-76 and 195-214) [16]. Although D25, a site 0 specific mAb, bound to the PR-TM, SC-DM, and SC-TM VLPs, as well as or better than the DS-Cav1 VLPs (Figure 3A), we considered the possibility that the results of sera blocking of mAb D25 binding to soluble F protein might be different using a soluble pre-fusion target that contained the SC and PR mutations. Figure 8, panel E, shows the results of serum antibody blocking of mAb D25 using soluble SC-TM as the target. The results were quite different from those obtained with the soluble DS-Cav1 target (panel B), indicating that the sera induced by the PR-TM and SC mutant F proteins contained a high concentration of antibodies that would block the binding of mAb D25 to a target containing the point mutations in site 0 and a lower concentration of antibodies that block binding to the DS-Cav1 target. By contrast, sera induced by DS-Cav1 VLPs could not block binding of mAb D25 to the SC-TM target (Figure 8E). 

As controls, we tested the ability of sera from the five VLP immunizations to block the binding of mAb AM14 and palivizumab to soluble SC-TM, although the five F proteins did not contain mutations at or near the sites recognized by these mAbs. Surprisingly, however, the results using soluble SC-TM as the target were quite different than the results using DS-Cav1 as the target. Figure 8, panels D and F, shows the results of serum antibody blocking of mAb AM14 and palivizumab binding, respectively, using purified the soluble SC-TM F protein as the target. Sera induced by DS-Cav1 VLPs and PR-DM VLPs did not block or only weakly blocked the binding of either mAb AM14 or palivizumab to the soluble SC-TM F protein target. The only sera that could effectively block the binding of mAb AM14 to the soluble SC-TM F protein target were induced by PR-TM, SC-DM, and SC-TM VLPs. Sera from all VLP immunizations could block the binding of palivizumab to the SC-TM target, to various degrees. These results suggest differences in the conformation of soluble DS-Cav1 and SC-TM or differences in the populations of antibodies induced by VLPs containing different pre-F proteins.

Interestingly, two sequential RSV infections yielded antibodies that would block mAb AM14 binding to the DS-Cav1 target moderately well, but did not block binding using the SC-TM target. This serum did not block D25 or palivizumab binding to either target. Thus, RSV infections yield populations of antibodies quite different from all the pre-F VLPs.

We considered the possibility that the failure of some sera to compete with mAb binding to the soluble SC-TM F protein was due to the failure of that sera to bind to this target adhered to Ni plates. A direct comparison of the total binding of each serum to the soluble SC-TM and soluble DS-Cav1 target showed binding to soluble SC-TM of sera from DS-Cav1 and PR-DM VLP sera was slightly less than binding to the soluble DS-Cav1 protein, while binding of sera from SC-DM and SC-TM to the two targets was the same (Figure S4, Supplementary Materials). However, the decrease in binding of some sera to soluble SC-TM seems unlikely to completely account for the lower levels or total absence of blocking mAb binding by some of the sera. Thus, the results of the blocking of binding to the soluble SC-TM F protein versus soluble DS-Cav1 are consistent with the idea that the populations of antibodies induced by PR-TM or the SC VLPs are different than those induced by DS-Cav1 VLPs.

### 3.7. Protection From RSV Challenge

**Figure 9:**
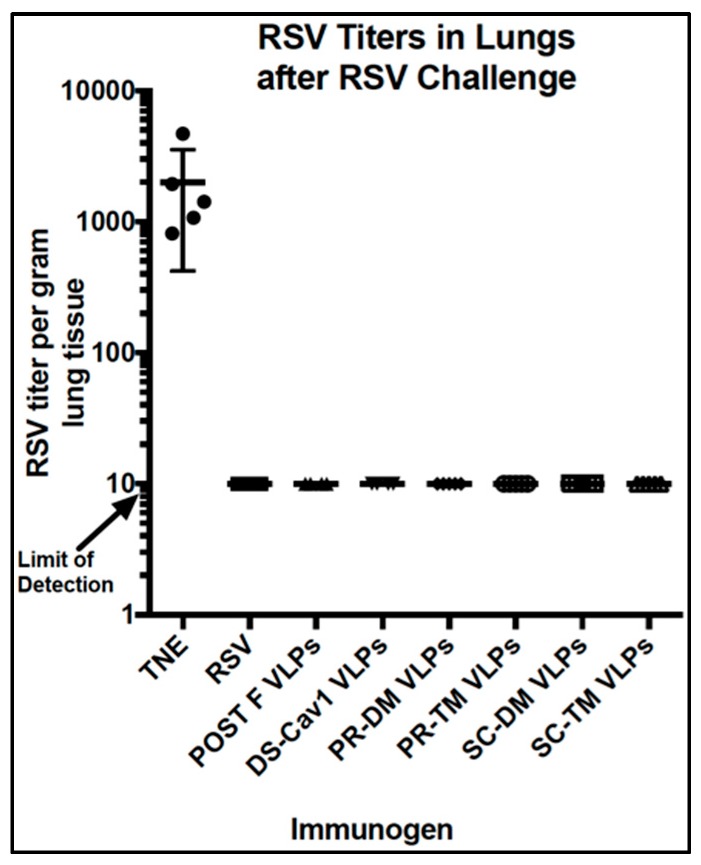
RSV replication upon RSV challenge of VLP Immunized mice. Mice were primed with VLPs or RSV infected at day 0 and boosted at day 100. At day 147, the mice were infected with RSV (1 × 10^6^ pfu/animal) and four days later, mice were sacrificed and the titers of RSV per gram of lung tissue were determined. No virus was detected above the detection limit for all immunized animals.

We next determined if there were differences in the protection of animals from RSV challenge after the different VLP immunizations. At seven weeks post-boost, immunized mice were RSV challenged and then sacrificed four days later. Lung titers were determined to assess any RSV replication after challenge. Figure 9 shows that there was no detectable virus in the lungs of any of the animals in any of the groups, except in sham vaccinated animals, a result that was expected since even a single RSV infection results in protection from RSV replication in mice [20,21]. Assessment of protection afforded by immunization with an RSV vaccine candidate is better done in cotton rats, which are more permissive to RSV infection.

## 4. Discussion

Following the groundbreaking studies of McLellan, et al, [16] who identified mutations in the RSV F protein (DS-Cav1 mutant protein) that stabilized the pre-fusion conformation, numerous laboratories and companies have reported the generation of alternative stabilized pre-fusion F proteins [27,34,35,36,37,38,39]. There have been only a few reports of systematic comparisons, other than total neutralizing antibody titers, of any differences between alternative versions of stabilized pre-fusion F proteins with respect to the properties of the protective antibodies they induce. Because the reported instability of the pre-fusion conformation of the soluble DS-Cav1 pre-fusion F protein could negatively impact the immune response to the protein, we explored the properties of alternative pre-fusion RSV F proteins. Krarup, et al. [27] have described a number of different mutations of the RSV F protein reported to stabilize the RSV pre-fusion F protein. For our study, four of these mutants were selected for the characterization of protein expression, the efficiency of assembly into VLPs, the stability of the pre-fusion conformation, the mAb reactivity of VLP associated F protein, and the induction of neutralizing antibody responses in mice, comparing the results with the VLP associated DS-Cav1 pre-F protein. In addition, the ability to block the binding of prototype monoclonal antibodies to protein targets by serum antibodies induced by each VLP was quantified. Taken together, the results are consistent with the conclusion that not all mutation stabilized RSV pre-F proteins have the same conformation or induce the same antibody responses. They do not induce similar levels of neutralizing antibodies or induce serum antibodies with similar antibody specificities.

A significant difference between the pre-F proteins was their levels of expression. The PR and SC mutants were expressed, on cell surfaces, at significantly higher levels than the DS-Cav1 F mutant and the post-F mutant. This finding suggested that the synthesis, folding, or intracellular transport of the PR and SC mutant proteins are more efficient than that of the DS-Cav1 F protein. This difference likely accounts for the different ratios of F and H/G and F and NP in the different VLPs.

The reactivity of the different VLPs to the pre-fusion specific anti-F protein monoclonal antibodies was surprisingly different. The PR-TM and SC-TM VLPs bound both mAbs D25 and AM14 at higher levels than DS-Cav1, indicating differences from DS-Cav1 VLPs. These differences may be due to altered accessibility of the mAb binding sites, by different affinities of the mAb to the different F proteins in VLPs, or differences in the conformation of the VLP associated F protein pre-fusion epitopes. By contrast, PR-DM bound both antibodies quite poorly and at much lower levels than DS-Cav1. These results suggest that the PR-DM mutant protein may be predominantly in a post-fusion conformation, or more likely in a conformation intermediate between the pre-F and post-F proteins. Monoclonal antibodies to sites in common with both pre- and post-fusion F, motavizamab, a site II antibody, and mAb1112 (site I), bound to all five pre-F VLPs at similar levels. However, there were some differences in the binding of the mAb 1243, a site IV antibody, again suggesting F protein conformational differences between the VLPs. 

There are three reports of the instability of soluble DS-Cav1 pre-F conformation [27,28,29] and, indeed, we have observed the loss of reactivity of this protein to mAb D25 upon storage. However, this protein, assembled into VLPs, was stable during incubation at high temperatures, high and low salt concentrations, high and low pH, and multiple cycles of freeze thaw, or upon prolonged storage. The pre-fusion conformation of the other four VLP associated pre-F proteins was also stable. Possibly, anchoring of the proteins in VLP membranes helps to stabilize the pre-fusion conformation. Additionally, the fusion of the ectodomains of these proteins to the NDV TM and CT domains and the inclusion of the foldon sequence at the carboxyl terminus of the F protein ectodomains may serve to stabilize the pre-fusion F protein conformation. 

Measures of neutralizing antibodies (NAbs) in sera showed that the PR-TM and PR-DM VLPs stimulated levels quite similar to those stimulated by the DS-Cav1 VLPs. Interestingly, the PR-DM VLPs were as effective as the DS-Cav1 and PR-TM VLPs in terms of the induction of NAbs in spite of the finding that the PR-DM VLPs bound pre-fusion specific mAbs D25 and AM 14 very poorly. We have previously reported that Post-F VLPs after both a prime and boost in mice stimulated about 1.5 to two-fold lower neutralization titers than DS-Cav1 VLPs [24]. Thus, the titers after PR-DM VLP immunization may reflect a mix of pre-F and post-F content in these VLPs. Alternatively, the PR-DM may be in a conformation intermediate between pre- and post-F, but a conformation that stimulates neutralizing antibodies. By contrast, SC-TM VLPs stimulated NAbs titers three-fold higher than DS-Cav1 VLPs, indicating that this version of the pre-fusion F protein more effectively stimulated NAbs in mice, a result consistent with the increased binding of mAb AM14 and D25 to these VLPs. SC-DM VLPs stimulated NAb levels twice those of DS-Cav1, consistent with the binding of D25 mAb to this VLP. This VLP may stimulate other pre-fusion specific antibodies not tested here.

There were no significant differences in the levels of total anti-pre-F or post-F-binding IgGs stimulated by the five different VLPs. This result suggests that different NAbs titers may be due to the different populations of specific antibodies in each serum. With the goal of defining potential differences in the populations of antibodies induced by the different VLPs, we quantified the amounts of anti-pre-F binding IgG required to block the binding of a given amount of monoclonal antibody to a pre-fusion target protein. Our results showed that the five-different pre-F VLPs induced quite different amounts of antibodies that blocked the binding of D25, AM14, or palivizumab to the target F protein. 

Complicating this analysis was the finding that the measured concentration of antibody that blocked mAb binding varied with the target F protein used. For example, the measured ng/mL of D25-blocking antibodies and AM14-blocking antibodies induced by DS-Cav1 VLPs was quite different when using soluble DS-Cav1 as the target compared to the values obtained using the soluble SC-TM protein target. These results further support the idea that alternative pre-F proteins induce different populations of anti-F antibodies. 

There are potentially several reasons for the ability of sera to block the binding of an mAb to a target protein. The polyclonal sera may have antibodies that bind directly to the epitope recognized by the mAb and thus they will directly block the binding of that mAb. However, mAb blocking may also involve relative affinities of the antibodies to the specific mAb binding site. It is likely that polyclonal antibodies with a lower avidity will not block binding as effectively as antibodies that have undergone affinity maturation. Indeed, at least for mAb D25, polyclonal antibodies from four and seven weeks post-boost in general blocked mAb binding better than antibodies at two weeks post-boost (Figure S3, Supplementary Materials). Differences could also relate to the affinities of the mAb to the target. For example, motavizumab is reported to have a higher affinity for site II than palivizumab [40]. This differential affinity could account for the observation that approximately 700 ng/mL of DS-Cav1 VLP sera was required to block the binding of motavizumab (Figure 7, panel C) to the DS-Cav1 target, while only 350 ng/mL of the same sera was required to block palivizumab binding (Figure 8, panel C). It is also possible that polyclonal antibodies in sera will be directed, not to the specific epitope recognized by the mAb, but to regions of the molecule in the vicinity of the epitope. Binding of these antibodies to off-site targets may block mAb binding to its site by masking the epitope, a concept described by Mousa, et al., for antibodies to site II [41]. Results of competition of any of the sera with the mAbs could be due to any or all of these possible mechanisms. 

Based on the combined results, particularly of mAb binding to VLPs, neutralization titers of sera in mice, and competition for binding of mAbs by sera induced by the five pre-F VLPs, the best antigen for inclusion in a vaccine is likely the SC pre-fusion F proteins, particularly SC-TM. The ultimate selection will depend upon the results of protection from RSV challenge. These studies are not informative in mice since even a single RSV infection results in the complete protection of mice from RSV challenge due, at least in part, to the limited permissiveness of mice to RSV replication. Thus, as expected, there was no detectable RSV in the lungs of any of the immunized mice four days after RSV challenge at day 147. Challenge studies, as well as assays for lung pathology after challenge, are better accomplished in cotton rats, which are quite permissive to RSV. Indeed, these studies, which are ongoing, clearly suggest significant differences in the protection provided by immunization with the different pre-F VLPs (in preparation).

## 5. Conclusions

The results indicate that not all mutation stabilized pre-fusion F proteins are the same. They indicate that different mutant stabilized pre-F proteins in VLPs have somewhat different conformations or accessibility of mAb epitopes and that there are differences in the levels and specificities of antibodies, as well as NAbs titers induced by these VLP associated pre-fusion F proteins. For selection of the appropriate form of the pre-fusion F protein for a vaccine candidate, these studies need to be extended to mice and cotton rats previously infected with RSV, a situation more closely mimicking the human population. Prior RSV infection (RSV priming) may well alter responses after different Pre-F VLP boost immunizations. 

## Figures and Tables

**Table 1:** Concentration (ng/mL) of Anti-Pre-F IgG Binding Antibodies That Block 50% of Binding of Monoclonal Antibodies to Soluble DS-Cav1 Protein.

Monoclonal Antibody		VLP Immunogen	
		Post-F	DS-Cav1 F
Am14	Exp 1	950 +/− 70	65 +/− 5
	Exp 2	675 +/− 170	95 +/− 7
	Exp 3	1500 +/− 100	138 +/− 56
D25	Exp 1	7500 +/− 4000	550 +/− 50
	Exp 2	2333 +/− 577	425 +/− 50
	Exp 3	8333 +/− 2100	505 +/− 130
Motavizamab	Exp 1	2000 +/− 100	825 +/− 25
	Exp 2	1000 +/− 50	1000 +/− 100

Shown are concentrations (ng/mL) of anti-pre-F binding IgG in pooled sera obtained at four weeks after boost immunizations with DS-Cav1 F VLPs or Post-F VLPs that blocked binding of mAb AM14, D25, or Motavizumab to the soluble pre-F protein target (DS-Cav1). Results are the mean of at least three separate determinations, with standard deviations indicated. Each group in each experiment (Exp) contained five mice. Values at or somewhat above 2000 ng/mL indicate sera that only very weakly blocked binding. Values at or above 2000 ng/mL were quite variable from experiment to experiment, as indicated by the large standard deviation.

## Supplementary Materials

The following are available online at http://www.mdpi.com/2076-393X/7/1/21/s1, Figure S1: Western blot of protein content of VLPs prior to adjustment for equivalent amounts of F protein. Figure S2: Relative binding of representative mAbs to purified VLPs. Figure S3: Blocking of binding of mAb by sera harvested at different times. Figure S4: Binding of sera induced by DS-Cav1 or SC-TM VLPs to the soluble DS-Cav1 protein or soluble SC-TM protein. Table S1: Relative concentrations of VLP proteins in different VLP stocks.
