# Supplementary material for: Comparison of Immune Responses to Different Versions of VLP Associated Stabilized RSV Pre-Fusion F Protein
Source: Vaccines (Basel). 2019 Feb 15;7(1):21. doi: 10.3390/vaccines7010021 (PMC6466353; doi:10.3390/vaccines7010021)
Supplement: Supplementary file 1 [file vaccines-07-00021-s001.pdf]

# Supplementary Materials

## Comparison of Immune Responses to Different Versions of VLP Associated Stabilized RSV Pre-Fusion F Protein

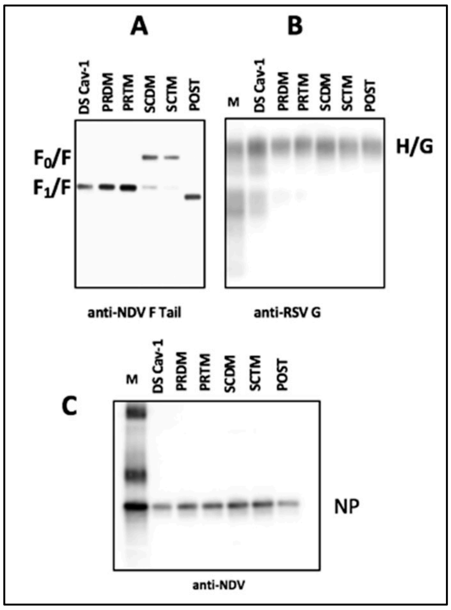


**Figure S1.** Western blot of protein content of VLPs prior to adjustment for equivalent amounts of F protein. Initial Characterization of VLP Protein Content: Figure shows VLP stocks of purified VLPs prior to final normalization of F protein content. Panel A: F/F protein content of VLP stocks; panel B, H/G protein content; panel C, NDV NP protein content. F0, uncleaved F/F chimera; F1, cleaved F/F chimera; H/G, NDV HN/RSVG protein chimera; NP, NDV NP protein; M, marker proteins.

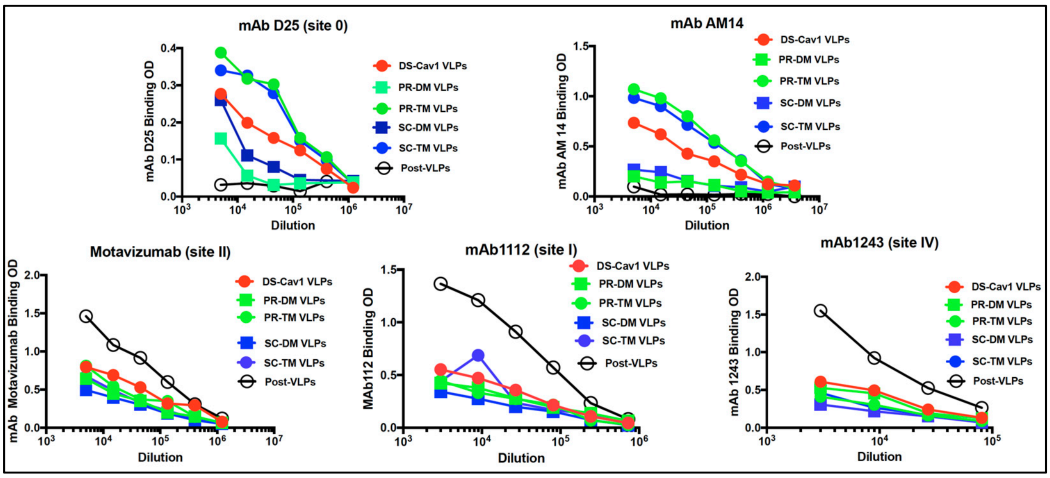


**Figure S2.** Relative binding of representative mAbs to purified VLPs. Relative binding of decreasing amounts of pre-fusion F specific monoclonal antibodies to VLPs is shown in panels A (mAb D25: site 0) and B (mAb AM14, a trimer specific antibody). Binding to VLPs of mAb specific to sites common to both pre- and post-fusion F proteins is shown in panels C-E. Panel C: motavizumab (site II); Panel D: mAb 1112 (site I); Panel E: mAb 1243 (site IV). Equivalent amounts of F protein in VLPs were bound to microtiter wells. Increasing dilutions of the antibodies were added to the wells and binding of the mAb was detected using anti-human (mAb D25, AM14, and motavizumab) or anti-mouse (mAb 1112, 1243) IgG coupled to HRP. Results are from a separate experiment to that shown in Figure 3.

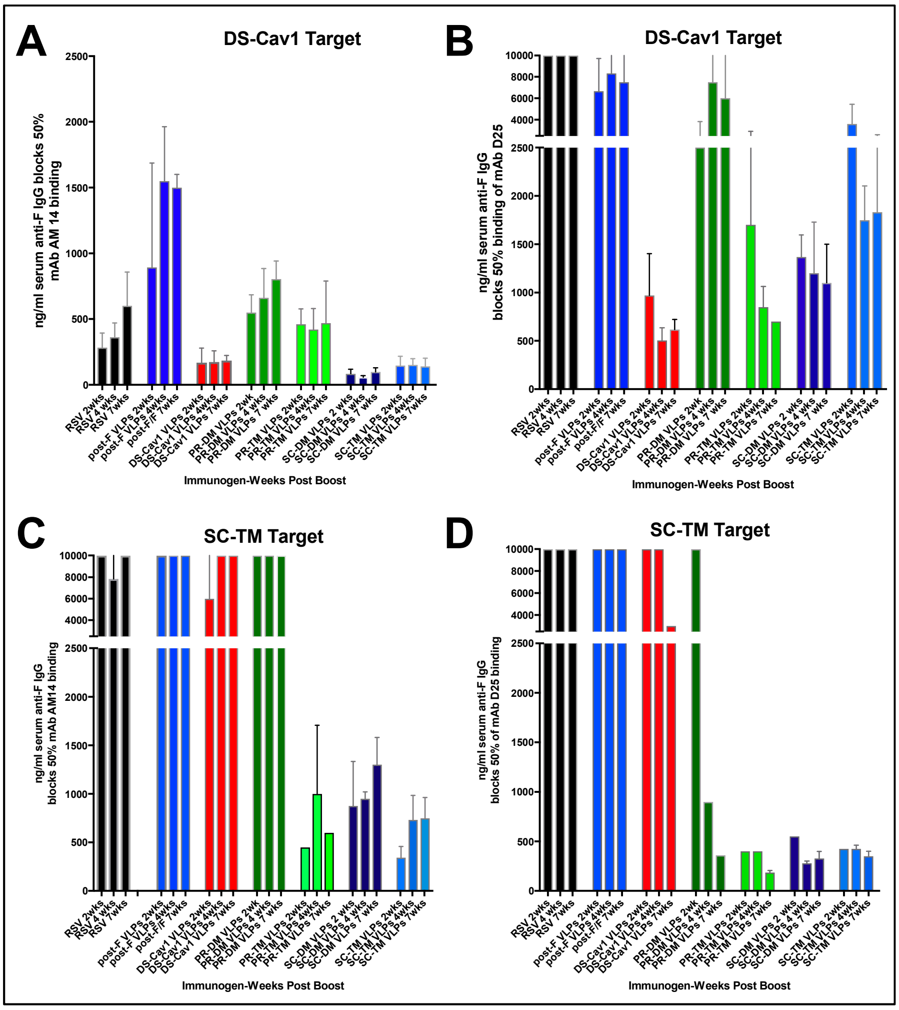


**Figure 3.** Blocking of binding of mAb by sera harvested at different times. Blocking of binding of representative mAbs by sera from PR, SC, and DS-Cav1 immunizations. Shown are concentrations (ng/ml) of anti-pre-F IgG in pooled sera obtained at two, four, and seven weeks after boost immunizations with all pre-fusion F VLPs, post-F VLPs, and RSV infection that block 50% binding of mAb AM14 (panel A) and D25 (panel B) to the DS-Cav1 target protein. Panels C and D show concentrations (ng/ml) of anti-pre-F IgG in pooled sera that block the binding of mAbs AM14 and D25, respectively, to the SC-TM target protein. The results are the mean of at least three separate determinations, with standard deviations indicated. Values at or near 10,000 ng/ml indicate failure of the sera to block binding of the tested mAb. Values at or above 2000 ng/ml indicate sera that only very weakly blocked binding. Values at or above 2000 ng/ml were quite variable from experiment to experiment, as indicated by the large standard deviation.

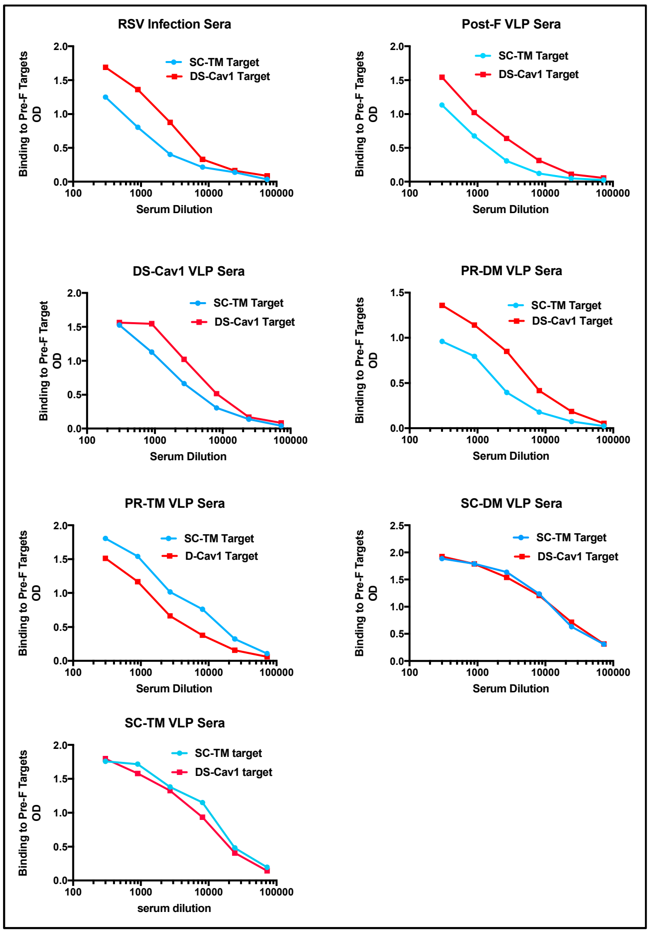


**Figure S4.** Binding of sera induced by DS-Cav1 or SC-TM VLPs to the soluble DS-Cav1 protein or soluble SC-TM protein. Binding of VLP induced sera to soluble DS-Cav1 and SC-TM targets. The binding of different dilutions of sera resulting from VLP immunizations or RSV infection to the DS-Cav1 target (red) or SC-TM (blue) target is shown.

**Table 1: Quantification of Proteins in VLPs**

| VLP | F/F protein anti-NDV F tail | F/F protein anti-RSV HR2 | H/G Protein anti-RSV | NP anti-NDV |
|---|---|---|---|---|
| DS-Cav1 | 1.0 | 1.0 | 1.0 | 1.0 |
| PR-DM | 1.11 | 1.0 | 0.6 | 0.44 |
| PR-TM | 1.30 | 1.10 | 0.71 | 0.43 |
| SC-DM | 1.21 | 1.13 | 0.77 | 0.60 |
| SC-TM | 0.92 | 0.83 | 0.5 | 0.46 |
| Post-F | 1.02 | 0.52 | 0.58 | 0.42 |

**Table S1.** Relative concentrations of VLP proteins in different VLP stocks. Concentrations of VLP proteins. The concentrations of F/F, H/G, and NP proteins in VLPs adjusted for equivalent F protein content are shown relative to the concentration in DS-Cav1 VLPs. Values for each protein were obtained by determining the density of the signal on Western Blots (Figure 2) (exposed in the linear range of detection), using Photoshop. The values for DS-Cav1 VLP proteins were set at 1.0 and values for proteins in the other VLPs are shown relative to DS-Cav1 values.
